# Pannexin 1 Regulates Dendritic Protrusion Dynamics in Immature Cortical Neurons

**DOI:** 10.1523/ENEURO.0079-20.2020

**Published:** 2020-08-20

**Authors:** Juan C. Sanchez-Arias, Rebecca C. Candlish, Emma van der Slagt, Leigh Anne Swayne

**Affiliations:** Division of Medical Sciences, University of Victoria, Victoria, British Columbia V8P 5C2, Canada

**Keywords:** cortical neuron, dendritic protrusions, dendritic spines, live imaging, neurodevelopment, pannexins

## Abstract

The integration of neurons into networks relies on the formation of dendritic spines. These specialized structures arise from dynamic filopodia-like dendritic protrusions. It was recently reported that cortical neurons lacking the channel protein pannexin 1 (PANX1) exhibited higher dendritic spine densities. Here, we expanded on those findings to investigate, at an earlier developmental time point (with more abundant dendritic protrusions), whether differences in the properties of dendritic protrusion dynamics could contribute to this previously discovered phenomenon. Using a fluorescent membrane tag (mCherry-CD9-10) to visualize dendritic protrusions in developing neurons [at 10 d *in vitro* (DIV10)], we confirmed that lack of PANX1 led to higher protrusion density, while transient transfection of *Panx1* led to decreased protrusion density. To quantify the impact of PANX1 expression on protrusion formation, elimination, and motility, we used live cell imaging in DIV10 neurons (one frame every 5 s for 10 min). We discovered that at DIV10, loss of PANX1 stabilized protrusions. Notably, re-expression of PANX1 in *Panx1* knock-out (KO) neurons resulted in a significant increase in protrusion motility and turnover. In summary, these new data revealed that PANX1 could regulate the development of dendritic spines, in part, by controlling dendritic protrusion dynamics.

## Significance Statement

Dendritic spines are microscopic structures that allow for communication between brain cells. Previous work showed that pannexin 1 (*Panx1*) knock-out (KO) increases the density of dendritic spines, raising the possibility that PANX1 could regulate their formation and/or stability. To address this research question, here we studied the role of *Panx1* KO and rescue on dendritic protrusions, the dynamic precursors of dendritic spines, in immature developing neurons. We found that *Panx1* KO increased the density and stability of protrusions on developing neurons, and conversely, that PANX1-EGFP expression decreased protrusion density, and increased protrusion turnover and overall movement. These results enhance our understanding of PANX1 regulation of neuronal development and neuroplasticity.

## Introduction

Although the mechanisms controlling plasticity of established dendritic spines are relatively well-characterized (Holtmaat et al., 2005; Araya et al., 2014; Sala and Segal, 2014; Schätzle et al., 2018), the molecular processes underlying their formation are less clear (Sando et al., 2017; Sigler et al., 2017; for review, see Südhof, 2018). Pannexin 1 (PANX1) is a four transmembrane domain protein that forms channels permeable to ions and metabolites with various activation mechanisms and diverse (patho)physiological implications (for review, see Boyce et al., 2018; Chiu et al., 2018). PANX1 is broadly and highly expressed in the brain during early postnatal development (Ray et al., 2005; Vogt et al., 2005) and localized and enriched in synaptic compartments (Zoidl et al., 2007; Sanchez-Arias et al., 2019). Pharmacological and genetic disruption of PANX1 results in enhanced induction of long-term potentiation (LTP) and impaired induction of long-term depression (LTD) in the hippocampus (Prochnow et al., 2012; Ardiles et al., 2014; Gajardo et al., 2018). Given that PANX1-interacting cytoskeletal regulators, collapsin response mediator protein 2 (CRMP2) and actin-related protein 2/3 complex (ARP2/3), are known to control dendritic spine formation (Wicki-Stordeur and Swayne, 2013; Spence et al., 2016; Zhang et al., 2016; Xu et al., 2018), it was perhaps not surprising that *Panx1* deletion led to the formation of larger cortical neuron network ensembles and that this was due, in part, to increased cortical neuron dendritic spine density (Sanchez-Arias et al., 2019).

It was unclear from the previous study (Sanchez-Arias et al., 2019) whether the increase in dendritic spine density associated with *Panx1* knock-out (KO) was because of an increase in the formation and/or an increase in the stability of the processes that develop into dendritic spines. Moreover, the previous study did not address whether re-expression of PANX1 could “rescue” the impact of *Panx1* KO. Dendritic spines develop from long, thin, highly dynamic processes called “dendritic protrusions” that are motile (i.e., grow and retract), and either disappear relatively rapidly (on the order of tens of seconds to minutes) or “survive” and stabilize. The goals of the current study were therefore 2-fold: (1) to investigate the impact of PANX1 on dendritic protrusion density and dynamics in developing cortical neurons; and (2) to determine whether the impact of *Panx1* KO could be rescued by re-expression of PANX1.

Because dendritic protrusion development is more readily accessible to study *in vitro*, and these systems (e.g., primary neuronal cultures) recapitulate many features of spine development observed *in vivo* (Ziv and Smith, 1996; Dunaevsky et al., 1999; Prange and Murphy, 2001; Zuo et al., 2005), we established an approach to study dendritic protrusions in immature cortical neuron cultures. Dendritic protrusions are long, thin, and contain relatively little cytoplasm. Therefore, standard cytoplasmic fluorescent proteins are not ideal for studying dendritic protrusions in immature, developing neurons. To this end, we devised a novel approach using the transmembrane tetra-spanin CD9-10 (*Tspan29*) protein fused to the monomeric red fluorescent protein mCherry (mCherry-CD9-10) and imaged dendritic protrusion dynamics in living neurons over a 10-min time interval. To determine the impact of *Panx1* KO on dendritic protrusion density and dynamics, and also to determine whether any observed effects could be rescued by re-expression of PANX1, we transiently transfected wild-type (WT) and *Panx1* KO neuronal cultures with control EGFP or PANX1-EGFP (as well as mCherry-CD9-10) and analyzed dendritic protrusions in fixed and living neurons at 10 d *in vitro* (DIV10). We confirmed that loss of PANX1 led to higher dendritic protrusion density. As anticipated, rescue of PANX1 in *Panx1* KO cultures, and transient expression of PANX1 in WT cultures led to decreased dendritic protrusion densities. Moreover, *Panx1* KO was associated with increased dendritic protrusion stability. Finally, transient PANX1 expression in *Panx1* KO cultures significantly increased motility and turnover of dendritic protrusions. In summary, these new data revealed an inverse relationship between PANX1 expression levels and dendritic protrusion stability, suggesting that the loss of PANX1, either through genetic deletion or during developmental downregulation, results in increased cortical dendritic spine density, in part by stabilizing dendritic protrusions.

## Materials and Methods

### Experimental animals

All animal procedures were approved by the University of Victoria Animal Care Committee and performed in accordance with the guidelines set by the Canadian Council on Animal Care. Male and female postnatal day (P)0–P1 mice were used in this study. C57BL/6J mice were obtained from The Jackson Laboratory (#000664, Table 1). The global *Panx1* KO strain in this study was derived from the *Panx1*^Vsh^ null mice, originally generated by Valery Shestopalov (Dvoriantchikova et al., 2012). Note that this strain was developed using targeted mutagenesis on 129-embryonic stem cells (129-ESCs, 129 × 1/SvJ x 129S1/Sv) with C57BL/6J as the recipient strain and backcrossed for five generations by the original authors. Recent comparative genomic analysis revealed the original *Panx1*^Vsh^ null mice contained a loss of function passenger mutation in *Casp4* (*Casp11*), common to five 129-substrains (129 × 1/SvJ, 129S1/SvImJ, 129S2/SvPas, 129S6/SvEvTac and 129P3/J) and are predicted to have passenger mutations in these additional genes: *Mmp1a*, *Olfr832*, *Fbxl12*, *ENSMUSG00000095186*, and *ENSMUSG00000095891* (Vanden Berghe et al., 2015). These mice were further backcrossed in-house onto a C57BL/6J for at least six generations (Sanchez-Arias et al., 2019). Mice were housed under a 12/12 h light/dark cycle starting at 8 A.M., with food and water *ad libitum*; temperature was maintained between 20°C and 25°C and humidity at 40–65%.

**Table 1:** Key resources table

Reagent or resource	Source	Identifier	RRID
Experimental models: organisms/strains			
Cortical neuron cultures from P0 C57BL/6J	The Jackson Laboratory	Catalog #JAX:000664	RRID:IMSR_JAX:000664
Cortical neuron cultures from P0 *Panx1* KO on a C57BL/6J background	(Dvoriantchikova et al., 2012;Sanchez-Arias et al., 2019)	NA	NA
Recombinant DNA			
mCherry-CD9-10	Addgene	Plasmid #55013	RRID:Addgene_55013
pEGFP-N1	Clontech (Takara Bio), discontinued	Catalog #6085-1	NA
*Panx1-*EGFP	(Penuela et al., 2007)	NA	NA
Chemicals, recombinant proteins			
DMEM/F12	Thermo Fisher Scientific		NA
NeuroCult	STEMCELL Tech.	Catalog #05713	NA
BrainPhys	STEMCELL Tech.	Catalog #05790	NA
Neurocult SM1	STEMCELL Tech.	Catalog #05711	NA
GlutaMAX	Thermo Fisher Scientific	Catalog #35050061	NA
P/S	Thermo Fisher Scientific	Catalog #15140122	NA
Gentamicin	MilliporeSigma	G1397	NA
Poly-D-lysine hydrobromide (PDL)	MilliporeSigma	P6407	NA
Dispase-1	MilliporeSigma	D4818-2MG	NA
Papain	MilliporeSigma	P4762-25MG	NA
DNAse-1	MilliporeSigma	11284932001	NA
Cytosine β-D-arabinofuranoside (ara-C)	MilliporeSigma	C1768	NA
Lipofectamine2000	Thermo Fisher Scientific	Catalog #11668027	NA
OptiMEM I	Thermo Fisher Scientific	Catalog #31985062	NA
Probenecid (water-soluble)	Thermo Fisher Scientific	Catalog #P36400	NA
My-Taq Extract PCR kit Vectashield	Bioline Vector Laboratories	BIO-21126 H-1000	NA RRID:AB_2336789
Software and algorithms			
FIJI (FIJI is just ImageJ)	NIH (Schindelin et al., 2012)	NA	RRID:SCR_002285
MultiStackReg v1.45 R Project for Statistical Computing (version 3.6.2)	Brad Busse (http://bradbusse.net/MultiStackReg1.45_.jar) The R Foundation	NA NA	NA RRID:SCR_001905
Rstudio	Rstudio Inc.	NA	RRID:SCR_000432
tidyverse package for R	CRAN (Grolemund and Wickham,2017)	NA	RRID:SCR_014601
dabestr package for R	CRAN (Ho et al., 2019)	NA	NA
Adobe Photoshop CS6 Leica Application Suite Software version 3.1.3.16308	Adobe Systems Inc. Leica Microsystems GmbH	NA NA	RRID:SCR_014199 RRID:SCR_013673
Equipment			
Leica TCS SP8	Leica Microsystems GmbH	NA	NA
8-well Nunc Lab-Tek Chamberedcoverglass PDL precoated coverslips	Thermo Fisher Scientific NeuVitro	155411PKGG- 12-PDL	NA NA

**Table 2:** Statistical table

	Figure	Comparison	Effect size [95CI]	Pairwise comparison	Type of test				
a1	1*B*	EGFP vs mCherry-CD9-10	37.7 [28.7; 44.9]	0.00384**	Student’s *t* test				
a2	1*C*	Observer # 1 vs Observer #2	0.95 [0.84; 0.98]	5.1e^−08^****	Pearson’s *R*^2^ correlation				
b1	2*Bi*	Dendritic protrusion density (#/10 µm) WT-EGFP vs *Panx1* KO-EGFP WT-EGFP vs WT-PANX1-EGFP WT-EGFP vs *Panx1* KO-PANX1-EGFP *Panx1* KO-EGFP vs *Panx1* KO-PANX1-EGFP	2.36 [1.28; 3.54] –3.24 [–4.54; –2.21] –3.72 [–5.17; –2.15] –6.08 [–7.84; –4.51]	0.03517* 0.00268** 0.00026*** 7.10e^−09^****	Two-way ANOVA with Bonferroni’s correction				
						df	Mean^2^	*F*	Pr(>F)
					Genotype	1	9.61	2.679	0.1095
					Plasmid	1	252.48	70.432	2.34e^-10^
					Interaction	1	22.05	6.151	0.0174
b2	2*Bii*	Dendritic protrusion length (µm) WT-EGFP vs *Panx1* KO-EGFP WT-EGFP vs WT-PANX1-EGFP WT-EGFP vs *Panx1* KO-PANX1-EGFP *Panx1* KO-EGFP vs *Panx1* KO-PANX1-EGFP	–0.13 [–0.397; 0.131] 0.105 [–0.21; 0.417] 0.031 [–0.31; 0.36] 0.161 [–0.178; 0.53]	>0.9999 >0.9999 >0.9999 >0.9999	Two-way ANOVA with Bonferroni’s correction				
						df	Mean^2^	*F*	Pr(>F)
					Genotype	1	0.1133	0.618	0.436
					Plasmid	1	0.2018	1.101	0.300
					Interaction	1	0.0085	0.046	0.831
c1	4*Bi*	Dendritic protrusion formation (%) WT-EGFP vs *Panx1* KO-EGFP WT-EGFP vs WT-PANX1-EGFP WT-EGFP vs *Panx1* KO-PANX1-EGFP *Panx1* KO-EGFP vs *Panx1* KO-PANX1-EGFP	–3.02 [–5.39; –0.803] 3.75 [–0.755; 10.3] 5.21 [1.21; 9.14] 8.23 [ 4.54; 11.8]	0.2267 >0.9999 0.2111 0.0028**	Kruskal–Wallis χ^2^ = 14.593, df = 3, *p* = 0.0022				
c2	4*Bii*	Dendritic protrusion elimination (%) WT-EGFP vs *Panx1* KO-EGFP WT-EGFP vs WT-PANX1-EGFP WT-EGFP vs *Panx1* KO-PANX1-EGFP *Panx1* KO-EGFP vs *Panx1* KO-PANX1-EGFP	–2.57 [–5.85; –0.209] 5.27 [0.455; 11.5] 9.06 [4.02; 13.8] 11.6 [7.5; 15.8]	0.62307 0.15616 0.00959 0.00024***	Kruskal–Wallis χ^2^ = 25.245, df = 3, *p* = 1.372e-05				
c3	4*Biii*	Dendritic protrusion lability (%) WT-EGFP vs *Panx1* KO-EGFP WT-EGFP vs WT-PANX1-EGFP WT-EGFP vs *Panx1* KO-PANX1-EGFP *Panx1* KO-EGFP vs *Panx1* KO-PANX1-EGFP	–1.11 [–3.17; 0.786] 2.34 [–0.966; 6.76] 6.2 [2.5; 9.81] 7.31 [3.92; 10.6]	>0.9999 >0.9999 0.0291** 0.0034**	Kruskal–Wallis χ^2^ = 13.421, df = 3, *p* = 0.00381				
c4	4*Biv*	Dendritic protrusion motility (%) WT-EGFP vs *Panx1* KO-EGFP WT-EGFP vs WT-PANX1-EGFP WT-EGFP vs *Panx1* KO-PANX1-EGFP *Panx1* KO-EGFP vs *Panx1* KO-PANX1-EGFP	–13.3 [–18.5; –8.42] 0.816 [–7.12; 9.33] –3.1 [–9.89; 3.97] 10.2 [4.53; 16]	0.00016*** >0.9999 >0.9999 0.03582*	Kruskal–Wallis χ^2^ = 20.442, df = 3, *p* = 0.0001374				
d1	5*Bi*	Dendritic protrusion survival fraction (%) WT-EGFP vs *Panx1* KO-EGFP WT-EGFP vs WT-PANX1-EGFP WT-EGFP vs *Panx1* KO-PANX1-EGFP *Panx1* KO-EGFP vs *Panx1* KO-PANX1-EGFP	2.21 [0.0663; 5.03] –4.4 [–9.04; –0.308] –8.19 [–12.3; –3.65] –10.4 [–14; –6.58]	0.81748 0.2034 0.00909** 0.00028***	Kruskal–Wallis χ^2^ = 24.351, df = 3, *p* = 2.11e-05				
d2	5*Bii*	Dendritic protrusion turnover (%) WT-EGFP vs *Panx1* KO-EGFP WT-EGFP vs WT-PANX1-EGFP WT-EGFP vs *Panx1* KO-PANX1-EGFP *Panx1* KO-EGFP vs *Panx1* KO-PANX1-EGFP	–4.48 [–7.73; –2.01] 6.68 [0.967; 14.7] 8.07 [2.84; 13] 12.5 [8.06; 17]	0.0092** 0.5205 >0.9999 0.0027**	Kruskal–Wallis χ^2^ = 19.895, df = 3, *p* = 0.0001784				
d3	5*Biii*	Dendritic protrusion Δmovement (%) WT-EGFP vs *Panx1* KO-EGFP WT-EGFP vs WT-PANX1-EGFP WT-EGFP vs *Panx1* KO-PANX1-EGFP *Panx1* KO-EGFP vs *Panx1* KO-PANX1-EGFP	–17.8 [–23.6; –11.9] 7.49 [–3.45; 20.1] 4.98 [–4.1; 13.8] 22.8 [15; 30.4]	6.2e-^05^**** >0.9999 >0.9999 0.00033***	Kruskal–Wallis χ^2^ = 28.526, df = 3, *p* = 2.816e-06				

Significance codes: ****<0.0001, ***<0.001, **<0.01, *<0.05.

### Primary cortical neuron cultures and transfections

Primary cortical neuron cultures were prepared as previously described (Sanchez-Arias et al., 2019). Briefly, cortices from male and female P0 pups from timed-pregnant WT and *Panx1* KO breeding pairs were microdissected and incubated with papain, dispase-1, and DNase-1 for 40 min in HBSS, followed by mechanical dissociation in DMEM/F12 medium supplemented with Neurocult SM1, GlutaMAX, and penicillin/streptomycin (P/S). A total of 125,000 cells were plated in a Nun Lab-Tek eight-well chambered coverglasses coated with PDL. One to two hours after plating, the medium was replaced with Neurocult supplemented with Neurocult SM1, GlutaMAX, P/S, and gentamicin. From DIV4 onwards, partial (half) medium changes were done with BrainPhys maturation medium (Bardy et al., 2015); to limit proliferation of glial cells, ara-C was added to the medium at DIV4. Transfections were performed at DIV6 using Lipofectamine2000. DNA/lipid complexes were diluted in OptiMEM-I at a ratio of 2 µg DNA:1 µl lipofectamine and incubated at room temperature for 30 min. Then, these DNA/lipid complexes were added to cells in BrainPhys medium without antibiotics and incubated for 1–1.5 h. Neurons were transfected with either pEGFP-N1 (250 ng) or *Panx1-*EGFP (250 ng, gift from Silvia Penuela and Dale Laird; Penuela et al., 2007). All transfections contained mCherry-CD9-10 (250 ng, a gift from Michael Davidson; Addgene plasmid #55013; http://n2t.net/addgene:55013; RRID:Addgene_55013) to visualize neurons and dendritic protrusions. All neurons in this study were used at DIV10. Neurons used for fixed quantifications were plated on PDL-coated coverslips.

**Figure 1.**
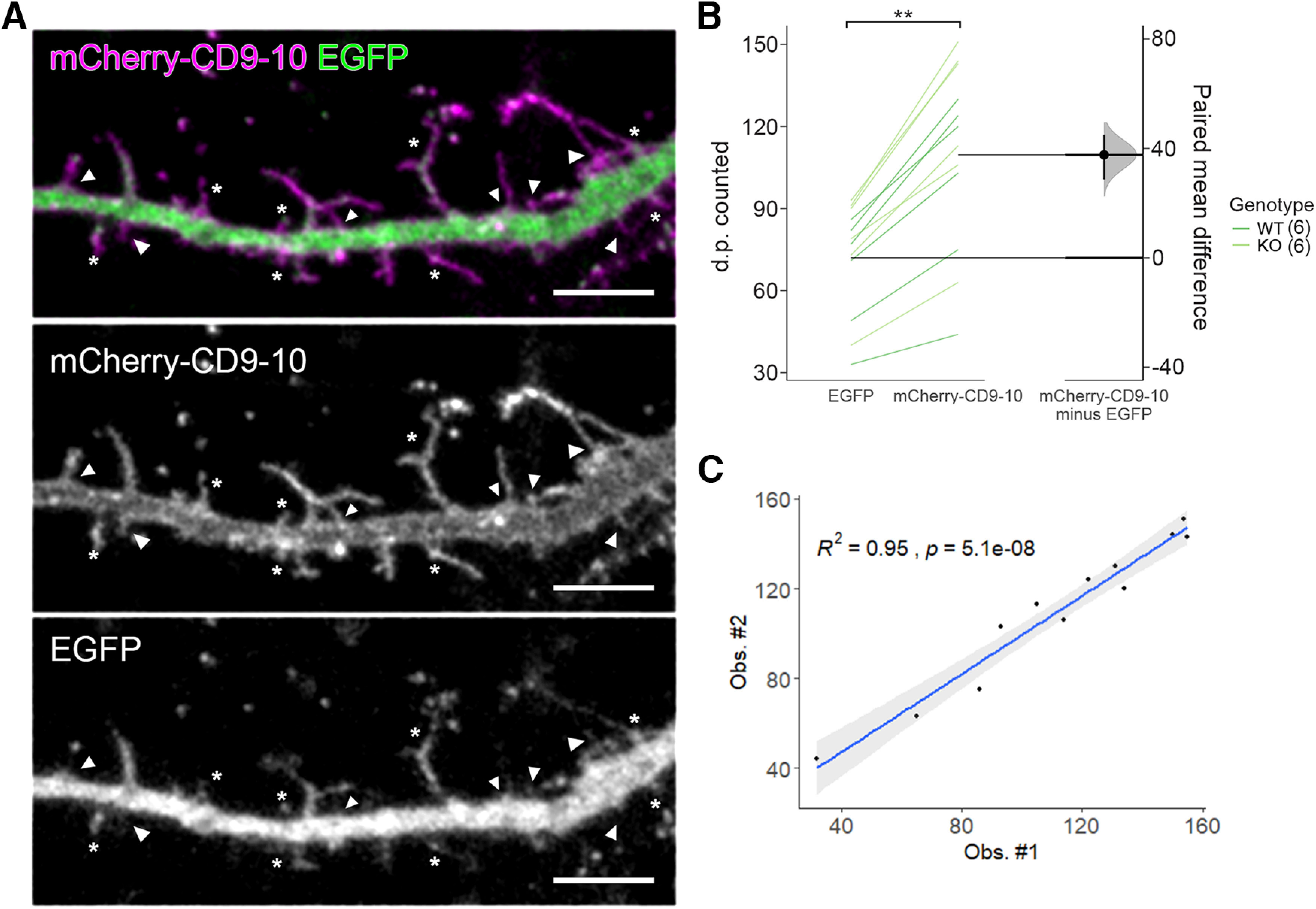
Detection of dendritic protrusions in cortical neurons was improved with a membrane-bound fluorescent marker. ***A***, Representative maximum intensity projection of a dendritic segment from a neuron transfected with mCherry-CD9-10 and EGFP at DIV6 and fixed at DIV10. Thin and long dendritic protrusions are more clearly visualized with mCherry-CD9-10 (mid) than the cytoplasmic volume marker EGFP (bottom). Structures not clearly labeled with EGFP are denoted by *, and those missed entirely are denoted with arrowheads. ***B***, Slopegraph showing the quantification of dendritic protrusions detected using the mCherry-CD9-10 signal compared with the EGFP signal. On average, 34% of dendritic protrusions detected with mCherry-CD9-10 were missed in the EGFP channel (EGFP: 72 ± 5.9 dendritic protrusion; mCherry-CD9-10: 110 ± 9.7 dendritic protrusions; *p *=* *0.00384, Student’s *t* test; *N* = 12 neurons, 6 WT and 6 *Panx1* KO^a1^). ***C***, The interobserver variability was evaluated with Pearson’s correlation and found to be *R*^2^ = 0.95 (95CI 0.84–0.98, *p *=* *5.1e^−08^)^a2^. d.p., dendritic protrusion; **<0.01. Effect sizes are reported in Table 2. Scale bar: 5 µm. These data are included in the PhD thesis of J.C.S.-A, University of Victoria (Sanchez-Arias, 2020), found at http://hdl.handle.net/1828/11714.

### Genotyping

A set of primers targeting upstream exon 3, exon 3, and downstream exon 4 of *Panx1* (CTTTGGCATTTTCCCAGTGT, CGCGGTTGTAGACTTTGTCA, and GTCCCTACAGGAGGCACTGA) were used to genotype mice as previously described (Sanchez-Arias et al., 2019). Genomic DNA was extracted from tail-clips using MyTaq Extract PCR kit. DNA from WT mice amplifies a single 585-bp band, whereas DNA amplified from global *Panx1* KO mice have a single 900-bp band.

### Imaging and analysis of dendritic protrusions in fixed cortical neurons

Dendritic protrusions (including filopodia) were defined as any membranous protrusions between 0.4 and 10 µm. Neurons were fixed on coverslips with 4% paraformaldehyde and 4% sucrose for 10 min and mounted on microscope slides with VectaShield antifade mounting medium. High-resolution images (3320 × 3320, pixel size: 0.088 µm, *z*-step size: 0.4 µm) were acquired using a Leica TSC SP8 microscope using a 40× immersion oil objective (1.30 NA) and exported to FIJI for analysis (Schindelin et al., 2012). Two experimenters were involved in dendritic protrusion density analysis. One experimenter quantified dendritic protrusions according to the inclusion criteria [neurons that expressed both mCherry-CD9-10 and EGFP/PANX1-EGFP, did not show signs of stress (blebbing, large vacuoles), and where dendritic protrusions were originating from the longest primary neurite] from all experimental groups. The other experimenter quantified dendritic protrusions only from mCherry-CD9-10 and EGFP-expressing neurons to obtain quantitative analysis of the improvement in dendritic protrusion detection realized with mCherry-CD9-10 versus EGFP signal and record interobserver variance in protrusion analysis. For quantitative assessment of dendritic protrusion detection realized with mCherry-CD9-10 versus EGFP signal, neurons were randomly selected by a second experimenter for analysis. Using the mCherry-CD9-10 signal, the experimenter traced individual dendritic protrusions emanating from the shaft of the longest neurite (primary neurite). The corresponding EGFP signal for each dendritic protrusion (initially traced using the mCherry-CD9-10 signal) was inspected and the presence or absence of a corresponding dendritic protrusion in the EGFP channel was recorded. The interobserver variability was determined by Pearson’s correlation and reported as *R*^2^. Dendritic protrusions from all experimental groups were traced in the same manner, and densities were calculated by dividing the total number of dendritic protrusions by the segment length and multiplying by 10 (dendritic protrusions per 10 µm). Representative images were processed uniformly with a Gaussian blur of 0.5 pixels, and uniform adjustments to levels and contrast were made using Photoshop CS6 Extended suite (Adobe Systems).

### Imaging and analysis of dendritic protrusions in live cortical neurons

Cortical neurons plated on chambered coverglasses and cultured in BrainPhys medium were placed in an incubation chamber attached to a Leica TSC SP8 microscope and held at 37°C and 5% CO_2_ for the entire duration of the experiment. After 15 min of acclimatization, primary and secondary dendrite segments within 100–150 µm from the cell body were imaged. We selected segments from the longest neurite (dendrite) that were clearly discernible from surrounding structures (other dendrites and cells). These segments ranged from ∼65 to ∼75 µm long. Time lapses of *z*-stacks (0.7-μm *z*-step size) were acquired every 5 s for 10 min at a resolution of 1024 × 256 (pixel size: 0.06 µm) using Leica’s TCS SP8 resonant mode (8000 Hz) and a 63× water immersion objective (1.20 NA). Images were exported to FIJI for analysis. First, the four-dimensionality (*x*, *y*, *z*, *t*) was reduced by creating maximum *z* projections before additional image processing, *x-y* drift was corrected with MultiStackReg v1.45 (developed by Brad Busse; http://bradbusse.net/MultiStackReg1.45_.jar) when required. Then, images were subjected to a low-pass filter using a Gaussian blur (kernel size 2) and thresholded using the *triangle* method (Zack et al., 1977; Polanco et al., 2018). From these binary images, outlines for each time frame were created and temporally color coded. Dendritic protrusions were manually counted, and four basic characteristics were recorded: formation, elimination, lability, and motility. We defined formation as any *de novo* appearance of a dendritic protrusion within the time-lapse recording; elimination was defined as the complete disappearance of a dendritic protrusion. Lability was defined as dendritic protrusions that were formed and eliminated within the duration of the time lapse, typically short-lived and lasting 1–3 min. To assess dendritic protrusion motility, we annotated partial extensions and partial retractions of individual dendritic protrusions. The survival fraction of dendritic protrusions was calculated by dividing the number of dendritic protrusions at the end of each time lapse (10-min mark) by the number of dendritic protrusions at the start (0-min mark). The overall turnover rate was calculated as the net percent gain and loss (sum of formation, elimination, and lability) of dendritic protrusions divided by the number of dendritic protrusions at the start of the time lapse. Lastly, the overall movement change of dendritic protrusions (Δmovement) was calculated by adding the fundamental characteristics of dynamics (formation, elimination, lability, and motility) divided by the number of dendritic protrusions at 0 min. Neurons that displayed signs of blebbing or for which focal planes were lost during the acquisition of images were excluded from analysis. Representative images were processed uniformly with a Gaussian blur of 0.5 pixels, and uniform adjustments to levels and contrast were made using Photoshop CS6 Extended suite (Adobe Systems Inc.).

### Experimental design and statistical analysis

Note that no additional experiments were completed after the initial submission because of the COVID-19 pandemic; these data are also included in the PhD thesis of J.C.S.-A (Sanchez-Arias, 2020). For experiments with fixed neurons, two coverslips from three independent cultures were used; for live cell imaging experiments, two to three eight-well chambered coverglasses from three independent cultures were used. Neurons from either WT or *Panx1* KO mice were plated in PDL-coated eight-well chambered cover glass or on PDL-coated coverslips in a randomized order by one experimenter, and all transfections, imaging, and image analysis were performed without knowledge of the genotype of the cultures by another experimenter. Neurons that displayed signs of blebbing or for which the focal plane was lost during image acquisition were excluded from analysis. Relevant details are described in Results, figure legends, and where appropriate, illustrated on the figures themselves. Data are presented as mean ± SD. Data analysis using bootstrap estimation (5000 bootstrap resamples), determination of effect size, bias-corrected confidence intervals, and Cumming estimation plots were generated using the dabestr package for R (Bernard, 2019; Calin-Jageman and Cumming, 2019; Ho et al., 2019). Null-hypothesis significance testing was performed using R (version 3.6.2), and *p* < 0.05 was used as the significant threshold for these tests. Normality was tested using the Shapiro–Wilk test (McDonald, 2014). Group analyses for normally distributed data were performed with a two-way ANOVA coupled to multiple comparisons with Bonferroni’s correction. For non-normally distributed data, Kruskal–Wallis pairwise comparisons with Bonferroni’s correction were used.

## Results

### Detection of dendritic protrusions in cortical neurons was improved with a membrane-bound fluorescent marker

Dendritic spines develop from highly dynamic dendritic protrusions. Genetic methods used to detect and measure these structures have mainly consisted of expression of cytoplasmic fluorescent proteins, such as EGFP. Transient transfection of EGFP (or one of its many variants) represent a convenient way to sparsely label neurons. There is a reasonable concern, however, that these cytoplasmic markers could fail to detect a substantial proportion of dendritic protrusions, given that these structures are relatively thin (and therefore contain a relatively small amount of cytoplasm; Fig. 1*A*). Membrane-bound lipophilic dyes (DiI, DiO, etc.), on the other hand, label thin membrane processes quite well (Mancuso et al., 2013). In order to combine the convenience of transient transfection of fluorescent proteins with the effectiveness of membrane-bound labels in thin process detection, we transfected cortical neurons with a fluorescent protein, mCherry, fused to a transmembrane protein, tetraspanin CD9-10 (mCherry-CD9-10, from Michael Davidson, Addgene plasmid #55013). As expected, the fluorescence signal from mCherry-CD9-10 delineated the plasma membrane (in addition to detecting somatic puncta most likely representing endosomes; Polanco et al., 2018). Qualitatively, it appeared that mCherry-CD9-10 improved the detection of dendritic protrusions. To quantify the improvement with mCherry-CD9-10, we traced dendritic protrusions using the mCherry-CD9-10 signal and compared this with the EGFP signal to confirm whether or not the same dendritic protrusions could be detected in that channel. We found that ∼34%^a1^ of dendritic protrusions detected using the mCherry-CD9-10 signal were not readily detected with the EGFP signal (Fig. 1*B*). We compared the number of dendritic protrusions detected between two observers using Pearson’s correlation and found them to be *R*^2^ = 0.95^a2^, suggesting mCherry-CD9-10 facilitated dendritic protrusion detection independent of the observer (Fig. 1*C*). These results suggest that mCherryCD9-10 represents an improved method for measuring dendritic protrusions in neurons.

### PANX1-EGFP expression rescued the increase in dendritic protrusion density associated with *Panx1* KO

**Figure 2.**
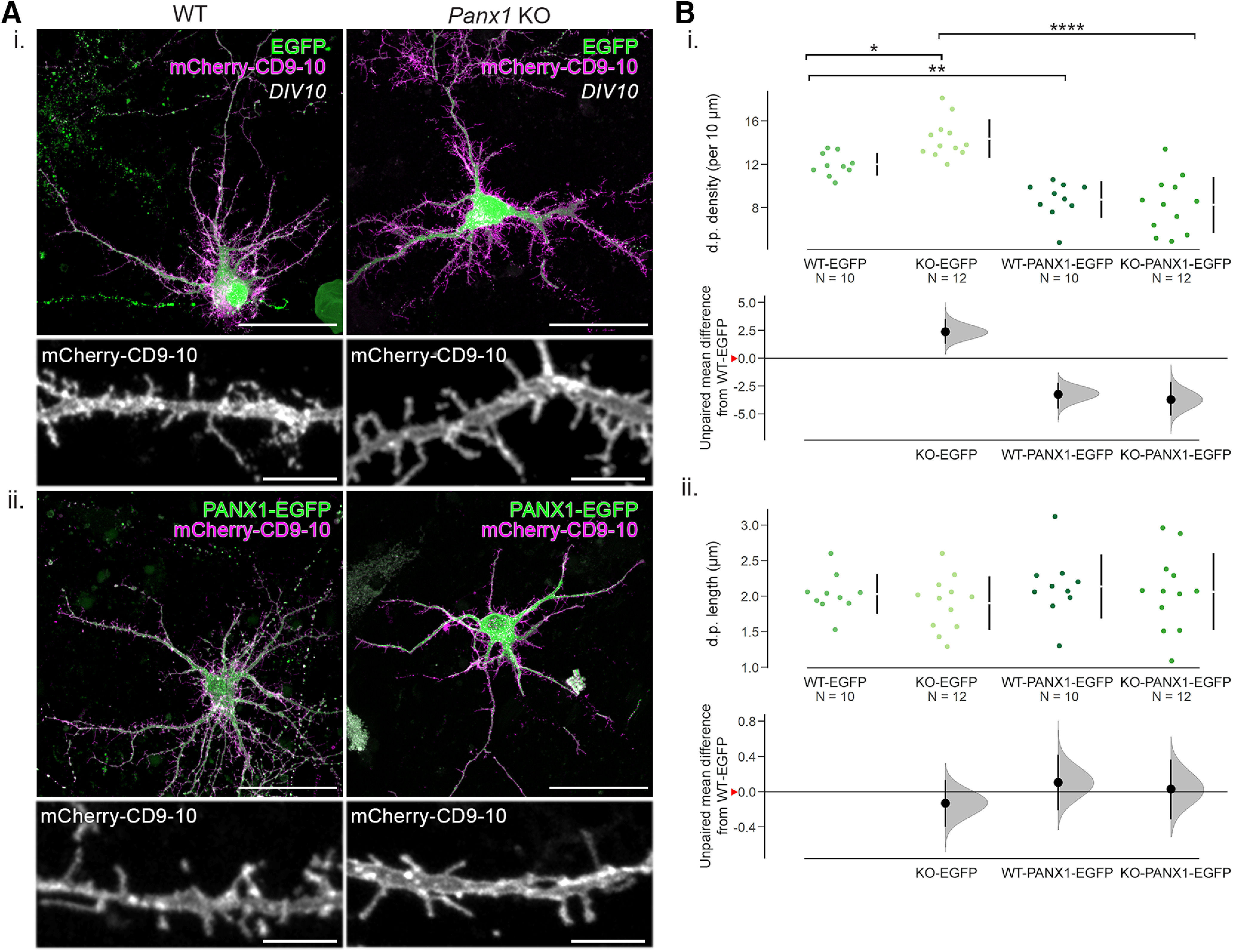
PANX1-EGFP expression rescued the increase in dendritic protrusion density associated with *Panx1* KO. ***A***, Representative maximum intensity projections of WT and *Panx1* KO cultured cortical neurons transfected with mCherry-CD9-10 and either EGFP (***Ai***) or PANX1-EGFP (***Aii***) as well as cropped images of their respective dendritic segments from a primary neurite. Scale bars: 50 and 5 µm. ***B***, Effect of PANX1 expression on dendritic protrusion density and length in developing cortical neurons transfected with mCherry-CD9-10 and either EGFP or PANX1-EGFP using Cumming estimation plots. ***Bi***, With EGFP expression, dendritic protrusion density was higher with *Panx1* KO neurons (WT-EGFP: 12.0 ± 0.3 dendritic protrusions per 10 µm; *Panx1* KO-EGFP: 14.4 ± 0.5 dendritic protrusions per 10 µm, *p *=* *0.03517, two-way ANOVA with Bonferroni’s multiple comparison test^b1^). With PANX1-EGFP expression, dendritic protrusion density was decreased in both WT and *Panx1* KO neurons (WT-PANX1-EGFP: 8.8 ± 0.5 dendritic protrusions per 10 µm, *p* = 0.00268; *Panx1* KO PANX1-EGFP: 8.3 ± 0.8 dendritic protrusions per 10 µm, *p *<* *0.0001, two-way ANOVA with Bonferroni’s multiple comparison test^b1^). ***Bii***, No significant differences in dendritic protrusion length were found between groups (WT-EGFP: 2.0 ± 0.3 µm; *Panx1* KO-EGFP: 1.9 ± 0.4 µm, *p *>* *0.9999, two-way ANOVA with Bonferroni’s multiple comparison test^b2^; WT-PANX1-EGFP: 2.1 ± 0.1 µm; *Panx1* KO PANX1-EGFP: 2.1 ± 0.2 µm, *p *>* *0.9999, two-way ANOVA with Bonferroni’s multiple comparison test). Data are presented as mean ± SD. *N* = cells, all analyzed cells were obtained from three independent cultures. Effect sizes are reported in the main text and Table 2. Red arrowheads on the *y*-axis on the bottom panel of Cumming estimation plots represent WT-EGFP means. d.p., dendritic protrusion; ****<0.0001, **<0.01, *<0.05. This figure was modified from the PhD Thesis of J.C.S.-A, University of Victoria (Sanchez-Arias, 2020), found at http://hdl.handle.net/1828/11714.

To investigate the impact of transient PANX1 expression on dendritic protrusion density in immature neurons, we transfected DIV6 WT and *Panx1* KO cortical neuronal cultures with mCherry-CD9-10 as well as EGFP (control) or PANX1-EGFP (overexpression for WT cultures, rescue for KO cultures). We fixed the cells 4 d later at DIV10 and measured the density of dendritic protrusions (Fig. 2*A*). In EGFP control-expressing cultures, we observed a 20% increase in dendritic protrusion density in primary neurites of *Panx1* KO neurons (effect size: 2.36 [95% confidence interval (95CI) 1.28; 3.54], *p *=* *0.03517,^b1^) compared with WT controls. In PANX1-EGFP-expressing cultures, we observed a 27% decrease in dendritic protrusion density in WT neurons (effect size: −3.24 [95CI −4.54; −2.21], *p *=* *0.00268^b1^; Fig. 2*Bi*) and a 42.5% density reduction in *Panx1* KO neurons (effect size: −6.08 [95CI −7.84; −4.51], *p* < 0.0001^b1^; Fig. 2*Bi*) compared with EGFP-expressing WT controls. Dendritic protrusion length was not significantly different among the groups (Fig. 2*Bii*^b2^). These results suggested dendritic protrusion density was inversely proportional to PANX1 expression levels.

### Novel methods for measurement of dendritic protrusions dynamics in living neurons

**Figure 3.**
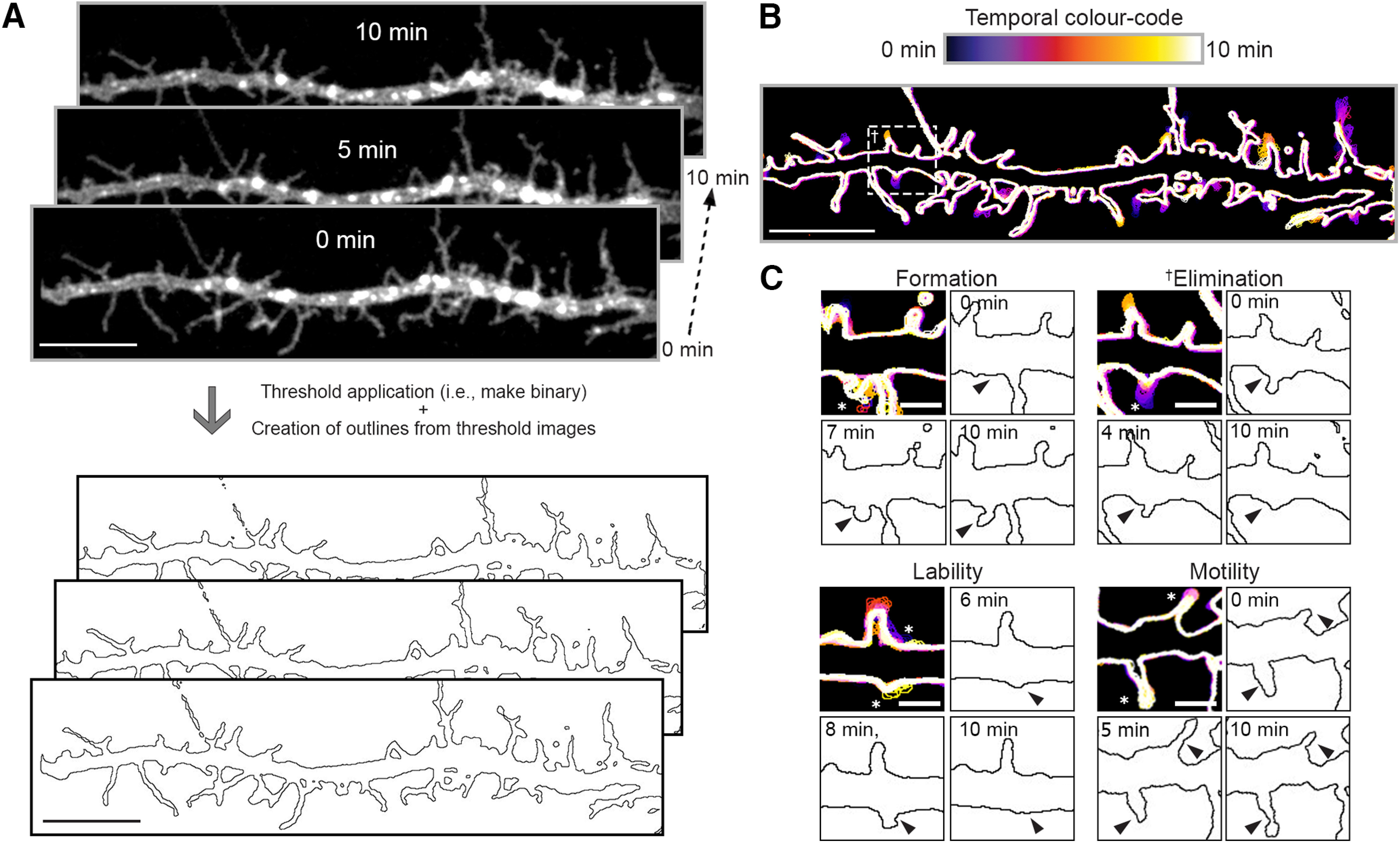
Novel methods for measurement of dendritic protrusion dynamics in living neurons. Ten-minute time lapses were acquired by imaging dendrite segments from cortical neurons every 5 s. Note that this a DIV10 WT cortical neuron transfected with mCherry-CD9-10 and PANX1-EGFP; only mCherry-CD9-10 is shown. The dimensionality of these recordings was reduced by creating maximum *z*-projections. Images were thresholded to create outlines (***A***; scale bar: 10 µm), which were temporally color coded (***B***; scale bar: 10 µm), allowing the visualization of various events, such as the percentage of dendritic protrusion (relative to time 0) undergoing formation (*de novo* appearance), elimination (complete disappearance by the end of the time lapse), lability (appearance and disappearance by the end of the time lapse), and motility (incomplete shrinkage or growth to an existing protrusion) shown in ***C*** (scale bar: 2 µm). Note that examples in ***C*** (cropped to highlight the event in question with (*) denoting the protrusion events) come from different cultures and different genotypes all at DIV10 transfected with mCherry-CD9-10 and either EGFP or *Panx1*-EGFP at DIV6. Note that the example provided here for dendritic protrusion elimination (**†**) in the box in part ***C*** comes from the larger neurite depicted in panel ***B***. Also note that the data shown in Figure 4 includes quantification from the examples depicted in this figure. For further details, see Materials and Methods. This figure was modified from the PhD thesis of J.C.S.-A, University of Victoria (Sanchez-Arias, 2020), found at http://hdl.handle.net/1828/11714.

To examine the mechanisms contributing to differences in dendritic protrusion densities between groups, we acquired 10-min time lapses (one frame every 5 s) of primary and secondary dendrites from cortical neurons at DIV10. These cultures were transfected with mCherry-CD9-10 and either EGFP or *Panx1*-EGFP at DIV6. At DIV10, dendrites harbor highly dynamic, thin, and long dendritic protrusions that are the precursors for dendritic spines (Ziv and Smith, 1996; Fiala et al., 1998). We reduced the dimensionality of the time lapses by creating maximum *z*-projections, and then images were passed through a low-pass filter and thresholded to create outlines (Fig. 3*A*). The dendritic silhouettes (Fig. 3*B*) were then temporally color coded to facilitate the detection of formation, elimination, lability, retraction, and growth of dendritic protrusions (Fig. 3*C*).

### Metrics of dendritic protrusion dynamics correlated with PANX1 expression levels

**Figure 4.**
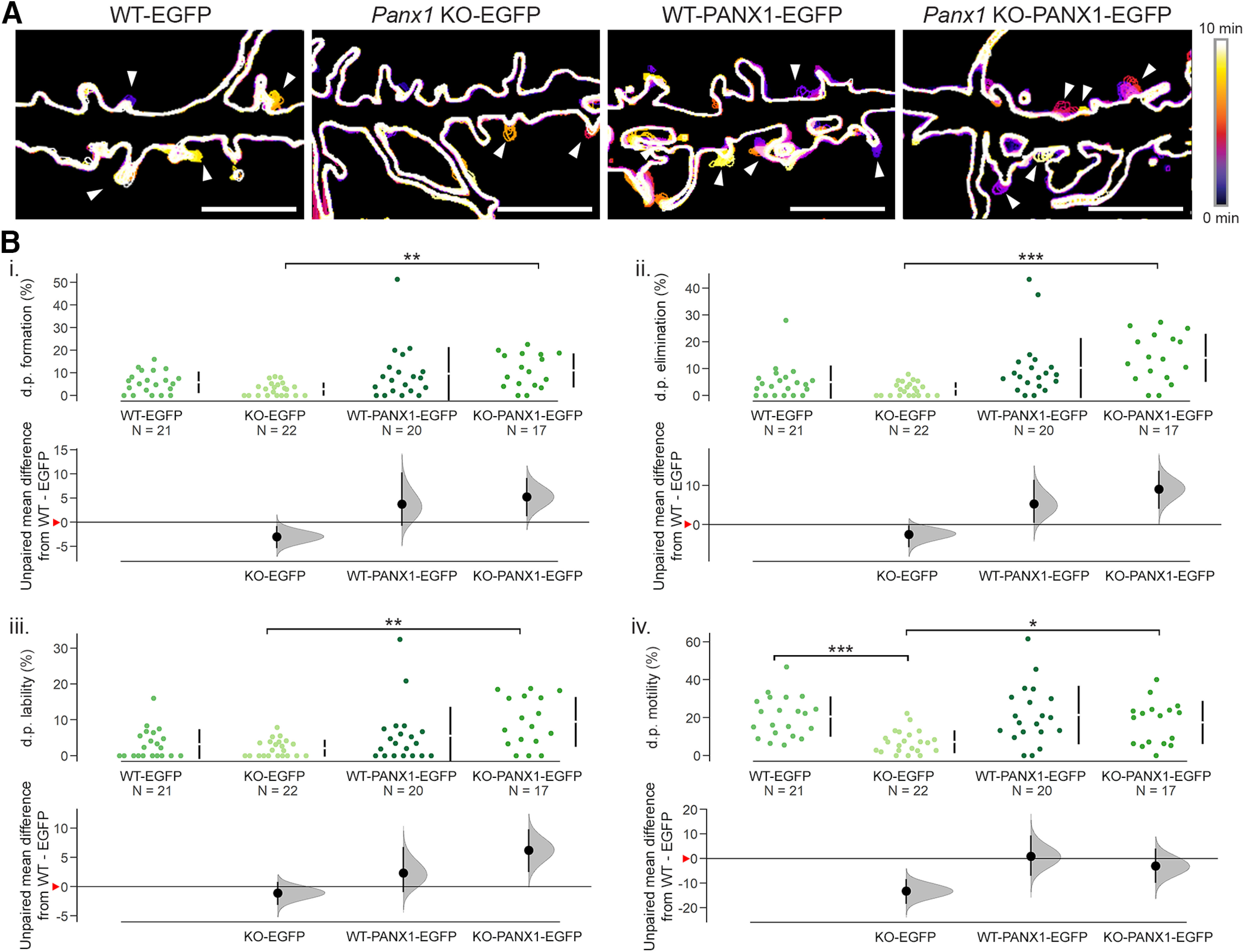
Metrics of dendritic protrusion dynamics correlated with PANX1 expression levels. ***A***, Representative color-coded outlines of WT and *Panx1* KO neurons transfected with mCherry-CD9-10 and either EGFP or *Panx1*-EGFP showing examples of dendritic protrusion formation, elimination, lability, and motility events (arrowheads). These examples are cropped from the full regions of analysis from primary neurites. ***B***, Effect of PANX1 expression on dendritic protrusion formation, elimination, lability, and motility in WT and *Panx1* KO using Cumming estimation plots. ***Bi***, Dendritic protrusion formation was significantly higher in *Panx1* KO neurons transiently expressing PANX1-EGFP compared with those expressing EGFP (KO-EGFP: 0.2 ± 0.1%, KO-PANX1-EGFP: 4.6 ± 1.3%, *p *=* *0.0028, Kruskal–Wallis test^c1^). No significant differences were observed between genotypes in EGFP-expressing neurons (WT-EGFP: 1.7 ± 0.7%; *Panx1* KO-EGFP: 0.2 ± 0.1%, *p *=* *0.2267, Kruskal–Wallis test^c1^). ***Bii***, Similarly, only transient expression of PANX1-EGFP in *Panx1* KO neurons increased dendritic protrusion elimination (KO-EGFP: 0.3 ± 0.15%; KO-PANX1-EGFP: 4.6 ± 1.28%, *p *=* *0.00024, Kruskal–Wallis test^c2^). No significant differences were found between EGFP and PANX1-EGFP expressing WT cells (*p *=* *0.62307^c2^). ***Biii***, Dendritic protrusion lability was higher in *Panx1* KO neurons transfected with PANX1-EGFP (KO-EGFP: 2.1 ± 0.5%; KO-PANX1-EGFP: 9.4 ± 1.7%, *p *=* *0.0034, Kruskal–Wallis test^c3^), beyond that observed in WT expressing EGFP control (*p *=* *0.0291, Kruskal–Wallis test^c3^). Transient expression of PANX1-EGFP in WT neurons had no significant effects (*p* > 0.9999^c3^). ***Biv***, Dendritic protrusion motility was significantly reduced in *Panx1* KO neuron expressing EGFP control (WT-EGFP: 20.5 ± 2.3%; KO-EGFP: 7.2 ± 1.3%, *p *=* *0.00016, Kruskal–Wallis test^c4^). Transient PANX1-EGFP expression increased dendritic protrusion motility in *Panx1* KO neurons only (KO-PANX1-EGFP: 17.4 ± 2.8%, *p *=* *0.03582, Kruskal–Wallis test^c4^). *N* = cells, all analyzed cells were obtained from three independent cultures. Effect sizes are reported in the main text and Table 2. Red arrowheads on the *y*-axis on the bottom panel of Cumming estimation plots represent WT-EGFP means. d.p., dendritic protrusion; ***<0.001, **<0.01, *<0.05. This figure was modified from the PhD thesis of J.C.S.-A, University of Victoria (Sanchez-Arias, 2020), found at http://hdl.handle.net/1828/11714.

Using our newly developed approach for the measurement of dendritic protrusion dynamics, we found that transient PANX1-EGFP expression significantly increased the formation (*de novo* appearance) and elimination (complete disappearance) of dendritic protrusions in *Panx1* KO neurons compared with transient EGFP expression (formation: effect size KO-EGFP vs KO-PANX1-EGFP: 8.23% [95CI 4.54%; 11.8%], *p *=* *0.0028^c1^; elimination: effect size KO-EGFP vs KO-PANX1-EGFP: 11.6% [95CI 7.5%; 15.8%], *p *=* *0.00024^c2^; Fig. 4*A*,*Bi*,*Bii*), while no significant differences were observed between PANX1-EGFP and EGFP expressing WT cultures (*p *>* *0.9999^c1^). Similarly, no significant differences were observed between genotypes in EGFP control-expressing cultures (formation: effect size WT-EGFP vs KO-EGFP: −3.02% [95CI −5.39%; −0.803%], *p *=* *0.2267^c1^; elimination: effect size WT-EGFP vs KO-EGFP: −2.57% [95CI −5.85%; −0.209%], *p *=* *0.62 307^c2^; Fig. 4*Bi*,*Bii*). We next quantified protrusion lability, a term we assigned to dendritic protrusions that transiently appeared and disappeared within 1–3 min (i.e., during our analysis period). Transient PANX1-EGFP expression significantly increased dendritic protrusion lability in *Panx1* KO neurons; whereas, there was significant impact of PANX1-EGFP expression in WT neurons (effect size KO-EGFP vs KO-PANX1-EGFP: 7.31% [95CI 3.92%; 10.6%], *p *=* *0.0034; effect size WT-EGFP vs WT-PANX1-EGFP: 2.34% [95CI −0.966%; 6.76%], *p* > 0.9999^c3^; Fig. 4*Biii*). WT and *Panx1* KO EGFP-expressing cultures exhibited no differences in dendritic protrusion lability (effect size WT-EGFP vs KO-EGFP: −1.11% [95CI −3.17%; 0.786%], *p *>* *0.9999^c3^). As anticipated, *Panx1* KO neurons exhibited significantly reduced dendritic protrusion motility (partial extension or retraction of an existing dendritic protrusion) compared with WT neurons within EGFP control-expressing cultures (effect size WT-EGFP vs KO-EGFP: −13.3% [95CI −18.5%; −8.42%], *p *=* *0.00016^c4^; Fig. 4*Biv*). Intriguingly, transient PANX1-EGFP expression increased dendritic protrusion motility in *Panx1* KO neurons only (effect size KO-EGFP vs KO-PANX1-EGFP: 10.2% [95CI 4.53%; 16%], *p *=* *0.03582^c4^). Together, these results suggest that dendritic protrusion movement roughly correlated with PANX1 expression levels.

### PANX1 increased dendritic protrusion turnover and overall movement

**Figure 5.**
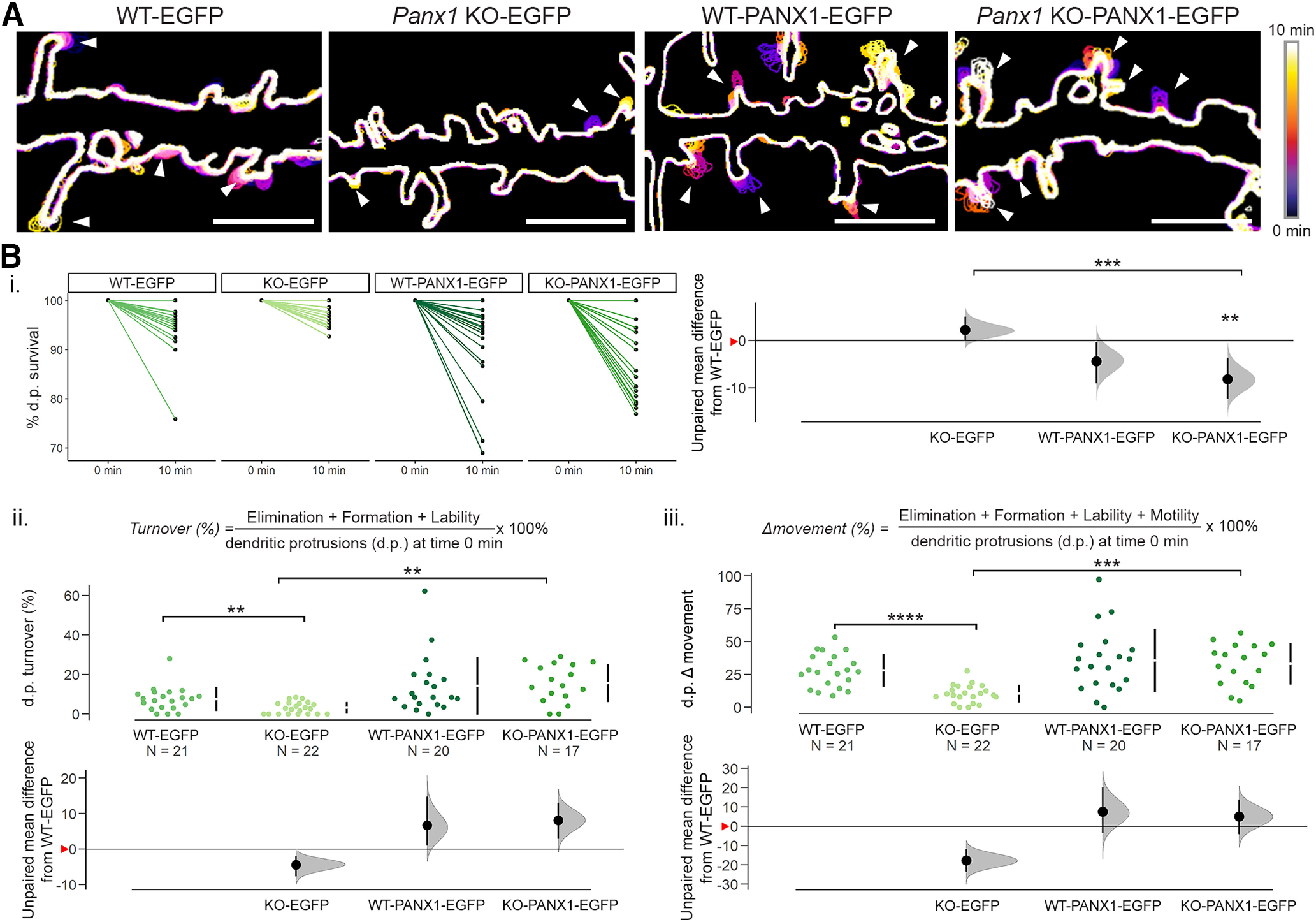
PANX1 increased dendritic protrusion turnover and overall movement. ***A***, Representative color-coded outlines of WT and *Panx1* KO neurons transfected with mCherry-CD9-10 and either EGFP or PANX1-EGFP showing examples of dendritic protrusion movement (arrowheads). These examples are cropped from the full regions of analysis from primary neurites. ***B***, Cumming estimation plots of dendritic protrusion second order metrics: survival fraction, turnover, and overall change in movement (Δmovement). ***Bi***, Transient PANX1 expression in WT and *Panx1* KO neurons decreased the survival fraction of dendritic protrusions; however, this was only statistically significant in *Panx1* KO neurons (WT-EGFP: 94.5 ± 1.2%; WT-PANX1-EGFP: 91.1 ± 1.9%, *p *=* *0.2034^d1^; PANX1-EGFP: 97.7 ± 0.5%; *Panx1* KO-PANX1-EGFP: 87.3 ± 1.9%, *p *=* *0.00028, Kruskal–Wallis test^d1^). ***Bii***, In the EGFP-control-expressing group, dendritic protrusion turnover was reduced in *Panx1* KO neurons (WT-EGFP: 7.5% ± 1.3; PANX1-EGFP: 3.1 ± 0.6%, *p *=* *0.0092, Kruskal–Wallis test^d2^). Transient expression of PANX1 significantly increased dendritic protrusion turnover in *Panx1* KO neurons but not in WT neurons (WT-PANX1-EGFP: 14.2 ± 3.3%, *p *>* *0.9999; *Panx1* KO-PANX1-EGFP: 15.6 ± 2.34%, *p *=* *0.0027, Kruskal–Wallis test^d2^). ***Biii***, Dendritic protrusion overall movement change (Δmovement) was reduced in *Panx1* KO neurons (WT-EGFP: 28 ± 2.8%; KO-EGFP: 10.3 ± 1.5%, *p* < 0.0001, Kruskal–Wallis test^d3^). PANX1-EGFP expression increased Δmovement in both WT (WT-PANX1-EGFP: 35.5 ± 5.4%) and *Panx1* KO neurons; however, this effect was only significant in *Panx1* KO neurons (KO-PANX1-EGFP: 33 ± 3.8%, *p *=* *0.00033, Kruskal–Wallis test^d3^). *N* = cells, all analyzed cells were obtained from three independent cultures. Effect sizes are reported in the main text and Table 2. Red arrowheads on the *y*-axis on the bottom panel of Cumming estimation plots represent WT-EGFP means. d.p., dendritic protrusion; ****<0.0001, ***<0.001, **<0.01. This figure was modified from the PhD thesis of J.C.S.-A, University of Victoria (Sanchez-Arias, 2020), found at http://hdl.handle.net/1828/11714.

To determine the overall impact of PANX1 expression on dendritic protrusion stability, we next used the fundamental metrics devised in Figure 3*C* to calculate dendritic protrusion survival, turnover, and overall change in movement (Δmovement). Survival, or the percentage of dendritic protrusions persisting at the end of the analysis period relative to time 0 min, was significantly reduced in PANX1-EGFP expressing neurons compared with EGFP control-expressing neurons within *Panx1* KO cultures only (KO-EGFP vs KO-PANX1-EGFP: −10.4% [95CI −14%; −6.58%], *p *= 0.00028^d1^; Fig. 5*A*,*Bi*). We next measured the overall gain and loss of dendritic protrusions (turnover) by adding together dendritic protrusion formation, elimination, and lability, divided by the total number of dendritic protrusions that were present at the beginning of the analysis (time 0). Turnover was significantly reduced in *Panx1* KO compared with WT neurons (effect size WT-EGFP vs KO-EGFP: −4.48% [95CI −7.73%; −2.01%], *p *=* *0.0092^d2^; Fig. 5*Bii*) in EGFP-expressing control cultures. PANX1-EGFP expression significantly increased turnover in *Panx1* KO cultures (effect size KO-EGFP vs KO-PANX1-EGFP: 5.24% [95CI 2.87%; 8.66%], *p *=* *0.0027^d2^). Finally, we added together the four fundamental metrics (formation, elimination, lability, and motility) to obtain an overall metric for the change in dendritic protrusion movement (Δmovement). Within EGFP control expressing cultures, Δmovement was significantly reduced in *Panx1* KO neurons compared with WT controls (effect size WT-EGFP vs KO-EGFP: −17.8 [95CI −23.6; −11.9], *p *<* *0.0001; Fig. 5*Biii*). Transient PANX1-EGFP expression resulted in increased Δmovement in *Panx1* KO cultures only (effect size KO-EGFP vs KO-PANX1-EGFP: 22.8 [95CI 15; 30.4], *p *=* *0.00033^d3^). Altogether, these results suggest that *Panx1* KO neuron dendritic protrusions are more stable and that this effect is rescued by transient expression of PANX1-EGFP.

## Discussion

### PANX1 increases dendritic protrusion movement

Previous work showed that *Panx1* KO was associated with an increase in dendritic spine density *in situ* and *in vitro* (Sanchez-Arias et al., 2019). However, it was unclear whether the increased dendritic spine density arose from additional formation and/or enhanced stability of spines. In order to investigate how PANX1 regulates the stability of dendritic protrusions, the precursors to dendritic spines, we developed a novel approach consisting of sparse expression of a membrane bound fluorescent protein, mCherry-CD9-10. This method significantly improved detection of dendritic protrusions over commonly used cytoplasmic fluorescent protein expression approaches (Mancuso et al., 2013). This novel methodology enabled us to discover a reciprocal relationship between PANX1 expression levels and dendritic protrusion density and stability. Taken together with the previous findings, this work suggests that PANX1 regulation of dendritic protrusion dynamics contributed to the increase in cortical dendritic spine density previously observed with *Panx1* KO. Moreover, these findings suggest that developmental downregulation of PANX1 likely contributes to increased spine stability underlying spine formation *in vivo* during the first four postnatal weeks of brain development.

Transient expression of PANX1-EGFP significantly affected dendritic protrusion dynamics in *Panx1* KO cultures but had less of an impact in WT cultures. The dampened impact of PANX1-EGFP in WT neurons implied the presence of a ceiling effect. Saturation of PANX1-mediated increases in dendritic protrusion movement may have resulted from limited availability of additional machinery for proper PANX1 trafficking and/or auto-regulation via ATP-dependent internalization (i.e., increase in PANX1 leading to increase in ATP release feeding back to internalize PANX1; Boyce et al., 2015; Boyce and Swayne, 2017). Alternatively, effects of supplementary PANX1 were perhaps constrained by limited availability of interacting partners or saturation of downstream purinergic signaling pathways (Abbracchio et al., 2009; Bhalla-Gehi et al., 2010; Wicki-Stordeur and Swayne, 2013; Dahl, 2015; Yang et al., 2015; Xu et al., 2018).

PANX1 regulation of dendritic protrusion stability could underlie neuronal network and synaptic plasticity changes associated with PANX1 disruption. For example, Rafael et al. (2020) recently reported that *Panx1* KO hippocampal neurons treated with tetrodotoxin (TTX) for 36 h (in a model of “chronic inactivity”) did not scale up their synapses accordingly, hinting that loss of PANX1 altered homeostatic plasticity mechanisms. Interestingly, the lack of synaptic scaling was seen both in *Panx1* KO neuron-glial co-cultures as well as *Panx1* KO neurons co-cultured with WT glia. On the other hand, WT neuron-glial co-cultures and WT neurons co-cultured with *Panx1* KO glia scaled up their synapses in the presence of chronic inactivity induced by TTX treatment. These findings suggest that neuronal, but not glial, PANX1 plays a role in the regulation of synaptic homeostatic plasticity and raises the possibility that increased stability of dendritic protrusions in *Panx1* KO could be a contributing factor to these altered synaptic plasticity adaptations (Holtmaat and Svoboda, 2009; O’Donnell et al., 2011; Yin and Yuan, 2015). Future studies should aim to elucidate the molecular mechanism underlying PANX1 regulatory role(s) in the maturation and maintenance of excitatory synapses throughout development and in the context of neuron-neuron (excitatory-inhibitory balance) and neuron-glial interactions (astrocytes, oligodendrocytes, microglia).

### PANX1 regulation of dendritic spines is consistent with its role in synaptic plasticity and neurodevelopment

Our findings provide important new insight into recent studies demonstrating that PANX1 regulates brain and cognitive development. *Panx1* KO has been associated with altered hippocampal plasticity (LTP facilitation and LTD deficits), impaired memory flexibility, and larger cortical neuron network ensembles and spine densities (Prochnow et al., 2012; Ardiles et al., 2014; Gajardo et al., 2018; Sanchez-Arias et al., 2019). Dendritic spine abnormalities have been observed in several neurodevelopmental disorders (Forrest et al., 2018; Lima-Caldeira et al., 2019). Brain enriched, autism spectrum disorders (ASDs)-associated single nucleotide polymorphisms (SNPs) have been associated with changes in *PANX1* expression levels (Davis et al., 2012). A germline *PANX1* SNP was associated with intellectual disability (Shao et al., 2016). PANX1 is also linked to neurodevelopmental disorders indirectly through its interacting partner CRMP2 and its downstream signaling effectors and interactors, purinergic receptors (Boyce et al., 2015; Boyce and Swayne, 2017; for review, see Swayne and Boyce, 2017). CRMP2 auto-antibodies have been implicated in ASD (Braunschweig et al., 2013), and brain-specific deletion of the gene encoding for CRMP2 resulted in decreased dendritic spine density and reduced LTP (Zhang et al., 2016), while suramin treatment corrected synaptic and behavioral phenotypes in the *Fmr1* KO mouse (Naviaux et al., 2013, 2015, 2017).

### Limitations

Immature neurons contain mainly filopodia-like structures and immature spines that lack fully developed postsynaptic densities (PSDs) (Berry and Nedivi, 2017). In order to facilitate observation of a substantial number of dendritic protrusions simultaneously, we selected a single immature time point (DIV10). We used this strategy because dendritic protrusions are the precursors to mature spines, although it should be noted that not all protrusions develop into spines. With this in mind, an important extension of the current study will be to assess synaptic markers within the same cells in a longitudinal approach (i.e., over multiple days), as this would enable tracking of dendritic protrusions that eventually develop into spines at a more mature DIV, as well as, retrospective grouping of our dendritic protrusion dynamics data based on the “fate” of the dendritic protrusions (i.e., attributes of those that become spines vs those that do not). Somewhat surprisingly, *Panx1* KO cortical neurons already exhibited increased dendritic protrusion density at this relatively immature analysis time point, suggesting that PANX1 begins to regulate dendritic protrusions, the precursors to dendritic spines, early in neuronal development. Previous work revealed a proportional increase in PSD-95-positive spines associated with *Panx1* KO at a more mature DIV (DIV12), suggesting the increase in dendritic protrusion density associated with *Panx1* KO indeed leads to a corresponding increase in dendritic spine density. In addition to facilitating tracking the fate of the dendritic protrusions, assessment of PSD-95 trafficking into spines, as a readout of maturation, would also permit simultaneous investigation of the mechanistic underpinnings of PANX1 regulation of dendritic spine formation. Spine formation relies on microtubule dynamics (Hu et al., 2011), and CRMP2, a PANX1 interacting protein, is a well-known regulator of microtubule dynamics as well as dendritic spine formation (Jin et al., 2016; Zhang et al., 2016). Additionally, it will be important to examine functionally similar proteins or mechanisms that could compensate for the loss of PANX1, such as changes in expression levels of other pannexins, functionally related connexins or purinergic receptors. Future pharmacological and genetic experiments disrupting PANX1 and its interactome will continue to enhance our understanding of PANX1 regulation of neuronal development.

There are some additional limitations associated with our KO model that are important to note. The original *Panx1^f/f^* strain was developed on a 129 × 1/SvJ background, and *Panx1* KO was generated via a cross with a *CMV*-*Cre* line (Dvoriantchikova et al., 2012), which were then backcrossed over 10 generations onto the C57Bl/6J background. The original strain contained a loss of function mutation in *Casp4* (*Casp11*) and was predicted to have variants in *Mmp1a*, *Olfr832*, *Fbxl12*, *ENSMUSG00000095186*, and *ENSMUSG00000095891* (Vanden Berghe et al., 2015). We cannot rule out the possibility that these passenger mutations could have been present in the global *Panx1* KO and not their control littermates; however, we also compared a conditional *Panx1 KO* (*Panx1^f/f^;Emx1^IRES-Cre^*) strain with control *Panx1^f/f^* littermates (i.e., same genetic background in conditional KO and control littermates), which yielded the same result in terms of dendritic spine densities, suggesting that this phenotype can be attributed to deletion of *Panx1*. Furthermore, transient expression of PANX1-EGFP rescued the change in dendritic protrusion density associated with *Panx1* KO, further supporting our findings from *Panx1* KO mouse that PANX1 inversely correlates with regulation of dendritic spine formation.

### Conclusions

In summary, this work has significantly advanced our understanding of the role of PANX1 in dendritic spine development, identifying it as a regulator of dendritic protrusion dynamics. PANX1 promotes dendritic protrusion movement and is associated with greater dendritic protrusion instability. The role of PANX1 in the regulation of this process that relies inherently on complex cytoskeletal dynamics is consistent with its previously identified interactions with ARP2/3 and CRMP2, known to control both cytoskeletal dynamics and dendritic spine development. Moreover, these novel findings presented here also align with the marked downregulation of cortical PANX1 expression levels in mice that coincides with spine formation during the first four postnatal weeks. Together, these findings reveal an important role for PANX1 in regulating dendritic protrusion dynamics in developing cortical neurons.

## Synthesis

Reviewing Editor: Douglas Bayliss, University of Virginia School of Medicine Decisions are customarily a result of the Reviewing Editor and the peer reviewers coming together and discussing their recommendations until a consensus is reached. When revisions are invited, a fact-based synthesis statement explaining their decision and outlining what is needed to prepare a revision will be listed below. The following reviewer(s) agreed to reveal their identity: Lucas Pozzo-Miller, Fenglian Xu.

Synthesis

This manuscript was seen by two reviewers, who recognize that the work nicely outlines the consequences of Panx1 deletion and overexpression on the density and dynamics of spine-like dendritic protrusions in cortical neurons in primary culture, and also refines use of a fluorescent membrane tag-mCherry-CD9-10 method for easily labeling the dynamic spiny membrane protrusions on dendrites. That said, a number of issues were identified that should be addressed, perhaps requiring additional experiments. These are outlined in the 3 major points from Reviewer #1. Reviewer #2 suggested additional studies to address interesting mechanistic possibilities, but the group consensus was that those could be saved for future work. Finally, you will find some suggestions for better focusing the Introduction and Discussion sections, particularly referencing other work on Panx1 in neuronal developmental or degenerative processes and emphasizing the advance provided by the very nice detection method used in this study.

Please find the full comments appended below.

----

Reviewer #1

Advances the Field

Solid results, somewhat confirmatory of prior published work, and need additional observations to increase the relevance of these new observations.

Comments

This manuscript reports the consequences of Panx1 deletion and its overexpression on the density and dynamics of spine-like dendritic protrusions in cortical neurons in primary culture. The manuscript is well written, and the data seem of sufficient quality to support the authors’ interpretations. However, a few issues may need to be addressed experimentally..

Major points

1. The authors need to estimate the percent of the dendritic protrusions that are (or will become) bona fide postsynaptic spines, by simultaneous imaging of a PSD marker and a presynaptic marker (either in fixed cultures or in time-lapse imaging with spectrally-shifted fluorescent proteins tagging pre and postsynaptic markers). Such additional data will enhance the significance of the work because it will provide direct evidence of the potentially differential role of Panx1 on bona fide postsynaptic spines, transient filopodia, or dendritic growth cones (that will become new dendritic branches).

2. What is the functional consequence of Panx1 deletion and overexpression on excitatory synaptic inputs? Such additional data could potentially reveal a differential effect on bona fide postsynaptic spines, transient filopodia, or dendritic growth cones, because only changes in postsynaptic spines would be reflected in the frequency of spontaneous mEPSCs, for example.

3. What was the statistical Power yielded by the sample numbers used in all the experiments (post-hoc Power analysis)? Also, the authors should explicitly state if cultures were randomly assigned to experimental treatments, and what criteria were used for data inclusion and exclusion (see Landis et al., Nature 2012).

Minor points

4. What is the percentage of glial cells in these cultures, and is it different in cultures from Panx1 KO mice or after Panx1 overexpression?

5. Do glial cells express Panx1? If so, what is the consequence of its deletion on their morphology/function and on the morphology/function of neighboring neurons?

Reviewer #2

Advances the Field

The major contributions of this study lie into two aspects:

1) Technical Advances: The authors optimized a fluorescent membrane tag-mCherry-CD9-10 method for effectively visualizing and analyzing thin spiny protections in primary cultured central neurons. This fluorescent tag method together with EGFP expression can beautifully label the dynamic spiny membrane protrusions on dendrites. The technique makes the study of these tiny and dynamic spiny structures easily.

2) knowledge Advances: This study shed new insight into how Panx 1 regulates the density of dendritic spines by regulating the dynamics such as the stability, motility, and turnover of membrane protrusions. This finding will stimulate future investigations of the molecular targets/cellular pathways that Panx 1 regulates dendritic spines.

Software Comments

There was a free online one mentioned, but I was not able to open in MacBook Pro.

Comments

This study provides novel, direct evidence that Pannexin 1(Panx 1) regulates the dynamics (motility, stability, and turnover) of dendrite spines in primary mouse cortical neurons (DIV 10). Specifically, the authors employed a novel, effective fluorescent membrane tag (mCherry-CD9-10) method to visualize thin spiny protrusions in fixed and living neurons derived from wildtype, Panx1 knock-out/rescue mice. Using this method in combination with EGFP, the group first confirmed previous findings that neurons from Panx-1 KO mouse exhibited higher spiny protrusion density and re-expression of Panx-1 in KO resulted in decreased density of spiny protrusion, indicating Panx-1 acts to down regulate spiny protrusions. They further revealed that in living neurons derived from Panx-1 KO animals, there was significant increase in stability and decrease in spine turnover. Rescue of Panx 1 in KO neurons leads to significant increase in the motility and turnover of spiny protrusions, leading to reduced density of spiny protrusions. The authors concluded that Panx1 expression has an inverse relationship with dendritic spine density due to its action on the dynamic structural motility and turnover.

Major concerns:

While the major finding is significant, the approach is novel and effective, and the manuscript is well written, the manuscript suffers from lack of mechanistical studies of how Panx 1 regulates the dynamics of dendritic spines. Without revealing a mechanism, it greatly diminished the impact of this study and reduced enthusiasm of readers.

In-depth studies may help answer the following questions and increase impact of the study:

1. Does Panx 1 interact with cytoskeletal proteins (e.g. actin) or scaffolding proteins (e.g. PSD95) to affect spine dynamics? Does Panx 1 directly interact, or indirectly interact via another molecule, or through factors released from Panx 1 channels? Such information is helpful in envisioning a potential Panx 1 working mechanism.

2. Is Panx 1 expression and/or function regulated by activity-dependent mechanism, considering that neuronal activity is a major causal factor involved in neuromorphogenesis, synaptogenesis and synaptic plasticity? In addition, Panx 1 has been linked to neuronal calcium waves and/or excitability changes via ATP/purinergic receptors and NMDA receptors, it would be interesting to explore if these (or other) transmitters, receptors, second messengers, and signaling pathways are involved in Panx 1 regulation of spine dynamics.

I understand that investigation of the underlying mechanisms could become an entire study for a separate manuscript, but it would be meaningful if the authors could at least provide more relevant literature discussion or speculations about Panx 1 regulation factors, signaling pathways, and molecular targets that might contribute to its action on spine dynamic regulation.

Minor

1) Both the Introduction and Discussion may need more work. More literature review is needed in the Introduction section to describe the known roles of Panx 1 expression on neuron spinal developmental or degenerative processes, as well as Panx 1-involved cellular, molecular and signaling pathways in neurons. This information helps the understanding about what has been studied, what is the gap knowledge, and what is significance of this study. Also, it would be useful to identify technical limits in studying spiny protrusions and thus increase the significance of novel detection method used in this study.

2) The current discussion emphasized heavily on clinical relevance, but the current study did not use diseased animal model. Instead, the authors could elaborate further on the potential working mechanisms as stated above.

3) Results Table 2 (Statistical Table) is overwhelming. I have had a hard time understanding this table, due partially to the difficulty in lining up the values and statistical significance to different experimental conditions. It would be better if this table can be condensed or be included in the supplemental material section.

4) Lastly, will Panx-1 impact immature, mature, and aging neurons in the same manners? Any regulation on inhibitory synaptic structures?
